# Therapeutic Targeting of Telomerase

**DOI:** 10.3390/genes7070039

**Published:** 2016-07-21

**Authors:** Kathrin Jäger, Michael Walter

**Affiliations:** 1 Institute of Laboratory Medicine, Clinical Chemistry and Pathobiochemistry, Charité-Universitätsmedizin Berlin, Augustenburger Platz 1, Berlin 13353, Germany; kathrin.jaeger@charite.de; 2 Labor Berlin-Charité Vivantes Services GmbH, Sylter Str. 2, Berlin 13353, Germany

**Keywords:** telomerase, telomeres, aging, senescence, atherosclerosis, cancer, gene therapy, immunotherapy, regenerative medicine, personalized medicine

## Abstract

Telomere length and cell function can be preserved by the human reverse transcriptase telomerase (hTERT), which synthesizes the new telomeric DNA from a RNA template, but is normally restricted to cells needing a high proliferative capacity, such as stem cells. Consequently, telomerase-based therapies to elongate short telomeres are developed, some of which have successfully reached the stage I in clinical trials. Telomerase is also permissive for tumorigenesis and 90% of all malignant tumors use telomerase to obtain immortality. Thus, reversal of telomerase upregulation in tumor cells is a potential strategy to treat cancer. Natural and small-molecule telomerase inhibitors, immunotherapeutic approaches, oligonucleotide inhibitors, and telomerase-directed gene therapy are useful treatment strategies. Telomerase is more widely expressed than any other tumor marker. The low expression in normal tissues, together with the longer telomeres in normal stem cells versus cancer cells, provides some degree of specificity with low risk of toxicity. However, long term telomerase inhibition may elicit negative effects in highly-proliferative cells which need telomerase for survival, and it may interfere with telomere-independent physiological functions. Moreover, only a few hTERT molecules are required to overcome senescence in cancer cells, and telomerase inhibition requires proliferating cells over a sufficient number of population doublings to induce tumor suppressive senescence. These limitations may explain the moderate success rates in many clinical studies. Despite extensive studies, only one vaccine and one telomerase antagonist are routinely used in clinical work. For complete eradication of all subpopulations of cancer cells a simultaneous targeting of several mechanisms will likely be needed. Possible technical improvements have been proposed including the development of more specific inhibitors, methods to increase the efficacy of vaccination methods, and personalized approaches. Telomerase activation and cell rejuvenation is successfully used in regenerative medicine for tissue engineering and reconstructive surgery. However, there are also a number of pitfalls in the treatment with telomerase activating procedures for the whole organism and for longer periods of time. Extended cell lifespan may accumulate rare genetic and epigenetic aberrations that can contribute to malignant transformation. Therefore, novel vector systems have been developed for a ‘mild’ integration of telomerase into the host genome and loss of the vector in rapidly-proliferating cells. It is currently unclear if this technique can also be used in human beings to treat chronic diseases, such as atherosclerosis.

## 1. Telomeres and Telomerase in Aging and Cancer

**Figure 1:**
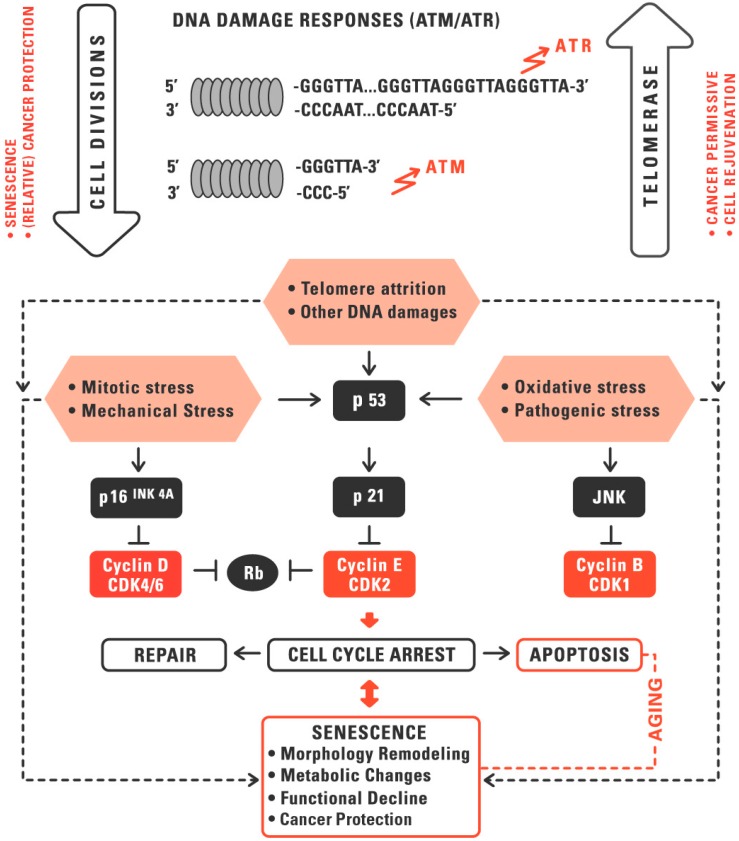
Replicative aging cooperates with other aging mechanisms to activate the p53 and/or Rb signaling pathways. ATM and ATR are sensors of DNA double- and single-strand damage induced by replicative senescence or other DNA damage. Activated ATM and ATR trigger checkpoint responses to induce cell cycle arrest. A stronger stimulation of p53 may lead to apoptosis by activating the mitochondrial pathway of apoptosis. Telomere length and cell function can be preserved by the reserve transcriptase telomerase, which synthesizes the new telomeric DNA from the RNA template. Telomerase may help to avoid senescence and to rejuvenate tissues, but is also permissive for carcinogenesis. Senescence may help to prevent tumor growth but can also be overcome by a process called crisis, and then has paracrine and other pro-tumorigenic effects. With permission, adapted from [1].

Aging is a complex process, which is accompanied by cycle arrest, remodeling in cell morphology and chromatin structure, functional decline, and extensive shifts in gene expression and metabolism. The aging of human cells can be mediated via stress-related mechanisms and via replicative senescence induced by telomere shortening. The various senescence triggers interact cooperatively and induce overlapping signaling pathways. Stressors include endogenous substances, exogenous factors, and species-specific mechanisms of aging such as replicative senescence. Replicative aging induced by telomere attrition is a species-specific aging mechanism, which acts as a tumor-suppressor in large, long-lived organisms [1] (Figure 1). Telomere attrition is, however, associated with functional decline and other negative effects that become relevant for the organism beyond the stone-age life span of approximately 50 years. An inverse correlation between telomere length and onset of age-related diseases has been shown in many studies even though the causality is still controversial [2].

Telomeres consist of repetitive non-coding DNA sequences (in humans TTAGGG), which are located at the end of the chromosomes. Telomeres, together with the shelterin complex, form a cap to protect the chromosome ends [3,4,5]. The shelterin complex consists of six telomere-associated proteins [6]. The telomere sequence is recognized by the subunits TRF1, TRF2, and POT1. These subunits are interconnected by the proteins TIN2, TPP1, and Rap1. The complex allows cells to distinguish telomeres from DNA damage sites. Without this protection, e.g., when telomeres shorten beyond a critical threshold, unprotected telomeres provoke a DNA damage response [7].

Telomere shortening occurs due to the so-called end replication problem, which means that the 3’ end of the DNA strand shortens with each cell division, since the DNA polymerase cannot completely replicate the strand [5,8]. At a certain threshold of telomere attrition the damage-repair system recognizes the unprotected DNA double strand as DNA breaks and activates the p53 or the p16INK4a signaling pathway to initiate a senescence or apoptosis program. Reactive oxygen species (ROS) or other environmental stress factors may also lead to telomere damage and accelerate the telomere attrition. Particularly, the GGG triplet within the human telomere sequence TTAGGG is vulnerable to chemical modifications. From a critical telomere length, onwards, telomeres are unable to claim the shelterin complex resulting in loss of the protective inner nucleotide loop, which ultimately leads to genomic instability [9,10] (Figure 1).

In numerous studies, it was observed that a healthy lifestyle is correlated with longer telomeres, likely reflecting protection against age-related diseases [4]. It has been shown in aging mice that cells with short and/or damaged telomeres are accumulating in stress-prone tissues, likely due to replicative exhaustion and/or stress-induced telomere damage. Animal studies suggest that senescence is not only a marker of, but also involved in, the propagation of age-related disorders [5,10].

From an evolutionary point of view, it is thought that the cell division limit was developed as a mechanism for tumor suppression. Indeed, in mice short telomeres are a hindrance to cancer growth. On the other hand, very short and damaged telomeres can also provoke tumor growth when a missing cap leads to chromosomal instability, as it is exemplarily observed in Dyskertosis congenita [11].

Telomere length and cell function can be preserved by the reserve transcriptase telomerase. The human telomerase consists of two subunits: a RNA templates (TERC, telomerase RNA component), and the catalytic subunit (hTERT, human telomerase reverse transcriptase), which synthesizes the new telomeric DNA from the RNA template [4]. Higher telomerase activities are under normal conditions only detectable in cells that need a high replicative capacity, such as stem cells and progenitor cells [9].

Telomere elongation by telomerase leads to chromosomal stabilization and a change to a more youthful gene expression pattern. In addition to its established role in extending telomeres, hTERT can promote proliferation of resting stem cells through a non-canonical pathway [9] and has direct effects on transcription and cell signaling, e.g., as a cofactor in a β-catenin transcriptional complex [12], which plays a role in embryogenesis and development [13]. Uncontrolled induction of telomerase would, however, have also pitfalls. Telomerase, per se, is no oncogene, but permissive for carcinogenesis and approximately 90% of all tumor cells express the enzyme to elongate the telomeres, which makes its use for systemic applications problematic.

The variety in telomere length in individuals of the same age is determined by genetic and environmental factors, leading to telomere damage and accelerated shortening of telomere length [14,15]. Inflammation is thought to contribute to telomere attrition in cells of the immune system by promoting leukocyte turnover and replicative exhaustion, and possibly also by direct modulation of telomerase activity by ROS and other stress factors [16]. For example, increased production of cytokines has been shown to adversely affect telomerase activity and telomere length [17]. C-reactive protein (CRP), a marker of inflammation, is inversely correlated with leukocyte telomere length (LTL) [18]. Shorter telomeres are associated with higher interleukin-6 (IL-6) and C-reactive protein values [19]. Telomerase activity was found to be reduced by psychological and life stress [20,21]. Various stressors trigger increased ROS formation, which leads to telomere attrition both directly and indirectly (by lower hTERT activity), which ultimately leads to a reduction of leukocyte telomere length (LTL) [4]. Smoking is an excellent example for higher ROS formation and, consequently, progressive shortening of telomeres [22].

At the cellular level, senescence serves as a natural tumor suppressor [23]. Senescent cells are no longer capable of replication and shut down their metabolism to a minimum. Only some key pathways are active and only few genes are expressed at higher levels. The senescence normally prevents the replication of abnormal chromosomes. The p16/pRb tumor suppressor pathways are activated in response to DNA damage and telomere dysfunction during senescence [23,24,25]. This process, however, could be flawed by oncogene activation to bypass senescence. An incorrect removal of senescent cells can lead to malignancy [23]. The alternative lengthening of telomeres (ALT) mechanism enables cancer cells with inactive telomerase the conservation of the telomere structure [26,27]. Approximately 5%–10% of cancer cells maintain their telomeres by ALT, in which sister chromatids exchange their telomeres by non-reciprocal recombination events [28]. Studies have shown that these cancer cells are more sensitive to ROS and drug treatments when they elongate their telomeres by ALT. Apparently these cells are under strong pressure to activate the alternative mechanism to escape senescence and apoptosis [26].

The catalytic subunit hTERT (human telomerase reverse transcriptase) was found to be upregulated in cervical carcinomas [29,30], hepatocellular carcinoma [31]], lung tumors [29], breast carcinomas [29], and neuroblastomas [29]. Telomerase in tumor cells is re-expressed on transcriptional, post-transcriptional, post-translational, and epigenetic levels [23]. Under normal conditions, the absence of CAAT and TATA elements in the TERT promoter prevents constitutive activation. However, promoter mutations or unusual epigenetic changes may overcome this barrier [32]. Telomerase also plays a regulatory role in the spread of cancer cells [33]. In the vast majority of investigated tumors an increased expression of human telomerase RNA (hTR) was detected, as well [34].

**Figure 2:**
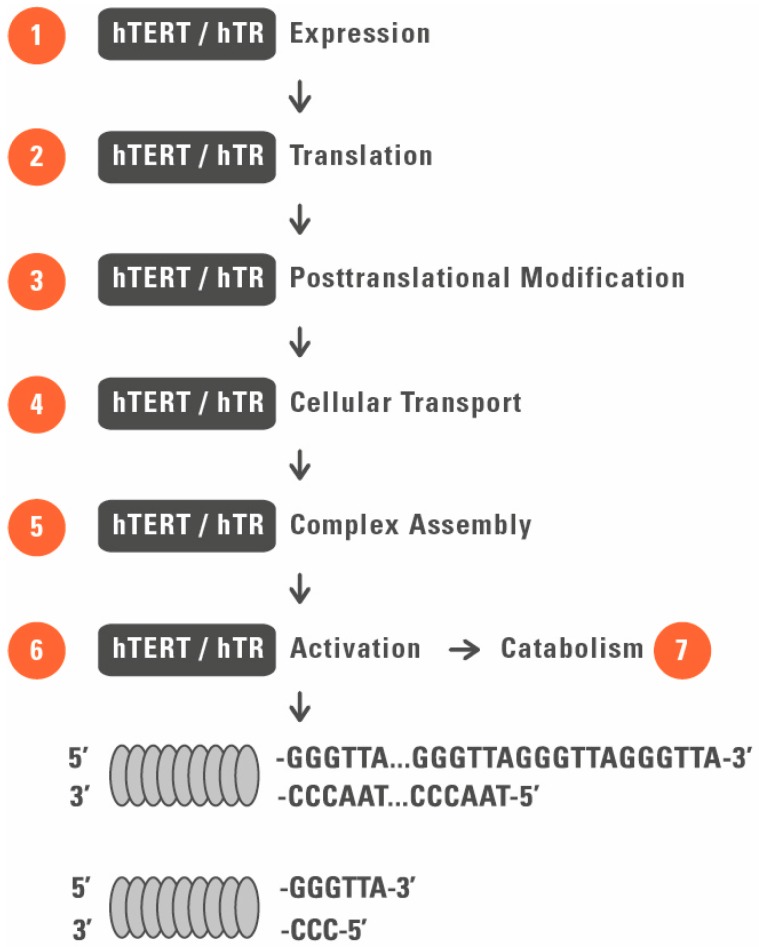
Therapeutic targeting of telomerase. Potential targets of telomerase for antitumor (telomerase suppressing) and rejuvenation (telomerase activation) drugs are shown by numbers 1–7. 1, inhibition/activation of gene transcription; 2, inhibition/activation of protein synthesis; 3, modulation of activity by posttranslational modifications; 4, modulation of telomerase activity by cellular sequestration; 5, interference with telomerase complex assembly; 6, modulation of signaling pathways and molecules involved in enzyme activation, such as Wnt/β-catenin, PI3K/Akt, and mTOR signaling; and 7, modulation of telomerase complex catabolism including vaccine therapy.

Telomerase is a target for both anti-cancer and cell rejuvenation strategies with a broad overlap of targets at different cellular and functional levels (Figure 1 and Figure 2).

## 2. Telomerase as a Target for Regenerative Medicine

Replicative senescence contributes to the decline in many physiological functions and in most tissues and, thus, contributes to the pathology of chronic diseases [35,36]. As telomerase activity is not, or only at low levels, detectable in somatic tissues there are many situations and chronic diseases in which the transient rejuvenation by telomerase immortalization could be a therapeutic option [16,37,38]. There are several possible strategies to reconstruct or enhance the enzymatic activity for therapeutic use:

*(1). Classical gene therapy with transfection of telomerase sequences:* This approach can be used for tissue engineering, for in vitro optimization of stem cell transplantation in donor cells with short telomeres [39] and, in principle, also for the treatment of chronic diseases in the whole organism, provided that induction of telomerase is time-limited.

*(2). Re-expression of silenced telomerase:* Cell differentiation normally leads to transcriptional downregulation of telomerase induced by signaling and epigenetic alterations [40,41]. However, telomerase downregulation can, at least in part, be reversed by various substances and mechanisms. Examples are histone deacetylase inhibitors [42] and estrogen receptor agonists, the latter acting by Akt mediated phosphorylation [43]. Many drugs with main targets other than telomerase also influence hTERT on transcriptional and/or posttranslational level. Involved signaling pathways that upregulate hTERT expression and/or activity (see also paragraphs below) are PI3/Akt, MAPK/ERK1/2, and the Wnt/β-catenin pathway.

*(3). Activation of residual enzymatic activity:* Activation of telomerase activity itself is an option for cells with residual telomerase activity such as stem cells of regenerative tissues and lymphocytes. In lymphocytes’ clonal expansion typically activates telomerase activity via enzyme phosphorylation and subsequent nuclear translocation [44]. This function declines with advanced age and leads to exhaustion of memory cells and could be restored by direct interaction with the telomerase holoenzyme or the telomerase activating signaling pathways [45].

*(4). Modulation of the intracellular location:* The sequestration of telomerase is another possible level of regulation on telomerase activity, implicating telomerase localization as a potential target for pharmacotherapy [46]. Telomerase can be translocated between the nucleus and the cytosol. hTERT is also present in mitochondria with yet unknown physiological significance [16,47].

Ectopic expression of telomerase was used to immortalize a wide variety of cell types including human fibroblasts [48,49,50,51,52], dermal fibroblasts [53,54], keratinocytes [55], muscle cells [56,57,58], vascular endothelial [59,60,61], myometrial [62], retinal [48,49,50,51,52], bone marrow stromal cells [63,64,65,66], osteoblasts [67,68,69], odontoblasts [70], CD4 and CD8 T cells [71,72], mesenchymal stem cells [72], myoblasts [73], hepatic stellate cells [74,75], fetal neuronal precursors [76], and breast epithelial cells [37]. Some cell types, such as bronchial and corneal cells, were used to form three-dimensional cultures [37].

Telomerase reconstruction was first discussed for treatment of diseases with distorted enzymatic activity of telomerase, namely, dyskeratosis congenital and aplastic anaemia [77]. Potential other applications are production of epithelia for burns or wounds, endothelia for blood vessels, chondrocytes for the treatment of arthritis, osteocytes for bone defects, and hematopoietic cells for bone marrow transplants or for the replacement of immune cells [39,78,79]. By use of this technique human blood vessels have already been engineered in vitro [56].

Transient telomerase activation may also be used for the treatment of other chronic diseases such as cardiac muscle disease, atherosclerosis [15], immunodeficiency, and bone marrow failure [11,80], liver disease [11,81], pulmonary fibrosis [11,82], degenerative cartilage defects [83], cataract [84], rheumatoid arthritis [85], organ transplantation [86], or treatments associated with the accelerated formation of senescent cells such as past cancer therapy or HIV [87,88]. Cartilage defects have become the target of cartilage tissue engineering [83]. Thomas and coworkers have demonstrated that bovine TERT-modified bovine adrenocortical cells can be transplanted into severe combined immunodeficient mice, and that these cell clones behave like their normal counterparts and form functional tissue after transplantation. This tissue is histologically similar to tissue formed from normal cells and shows a similar rate of cell division, implying a therapeutic role of telomerase in xenotransplantation [86].

The association between telomere length and aging has also led to the development of telomerase activators that may induce hTERT and/or hTR expression, enhance enzyme activity and/or influence cellular location. The idea behind this approach is to reverse normal cellular aging and to treat symptoms of aging. A single molecule telomerase activator, cycloastragenol (commercially available as TA-65, derived from *Astragalus membranaceus* root), has been shown to transiently activate telomerase in T lymphocytes [87], associated with the retardation of telomere shortening, increased proliferative potential, and enhanced functional response [88]. This substance was proposed to be used for the treatment of accelerated immunosenescence in HIV patients to increase the number of senescent memory CD8 T cells [87,88]. Cycloastragenol (TA-65) has been sold as a food supplement since 2013 and has been identified as an effective telomerase activator in immune cells, neonatal keratinocytes, and fibroblasts [87,89], acting via ERK-pathway activation and subsequent enhancement of telomerase expression. It increases the telomere length in mice without increasing the cancer incidence [90]. In a small pilot study it was used for the treatment of age-related macular degeneration [91] and was shown to improve markers of metabolic, bone, and cardiovascular health [92]. A moderate increase in leukocyte telomere length was shown in humans [93]. However, the number of patients in these studies was limited and some effects were of borderline significance. Long-term prospective studies regarding positive or side-effects are lacking.

Other phytochemicals have been shown to activate telomerase. Resveratrol activates telomerase in mammary epithelial [94] and endothelial progenitor cells [95], most likely due to the upregulation of SIRT1 [96]. Current knowledge regarding possible long-term effects is, also for this substance, incomplete [96]. For the treatment of cataracts pharmaceuticals with telomerase activating effects such as N-acetylcarnosine have been proposed since reduced telomere length is intimately involved in opacification, making the lens opaque or cloudy [84]. Another compound (AGS-499) has neuroprotective effects in mice and showed delayed progression of amyotrophic lateral sclerosis and increased survival in SOD1 transgenic mice [97]. Bone marrow mesenchymal stem cells often display premature aging und unstable proliferation. It has recently been shown in a rat model that co-transfection of BMSCs with telomerase and nerve growth factor had a better effect on learning and memory compared to cells lacking these factors [98]. These effects may be used for the development of therapeutic strategies to treat cognitive impairment in vascular dementia.

Indirect strategies to upregulate telomerase activity are described for certain antioxidants, such as N-acetylcysteine, which blocks the nuclear export of telomerase into the cytosol [99] and α-tocopherol, which was shown to retain telomerase activity in brain microvascular endothelial cells [100]. The idea behind this concept is that ROS damage telomeres directly (by damaging the vulnerable GGG triplet of the repetitive telomere sequence) and indirectly (by modulating telomerase activity and cellular location) [101]. HMG-CoA reductase inhibitors may also have telomere lengthening effects [102], by interfering with the redox balance of cells [99] and by increasing expression of the telomere stabilizing protein TRF2 [103]. Finally, Ginkgo biloba was shown to activate telomerase by inducing PI3K/Akt signaling [104].

*Telomerase upregulation in skin diseases:* The skin is an organ for which some therapeutic approaches of telomerase are already in use. Several therapeutic strategies have been proposed based on in vivo or ex vivo stimulation of stem/progenitor cells for expressing hTERT or telomere RNA component (hTR) and for the replacement of dysfunctional or lost skin [37,105,106,107,108,109,110,111]. The application area ranges from reconstructive surgery after severe wounds, burns, deep skin injuries, infections, and decubitus ulcers [106,107] to treatment of diseases with defects in hTERT and/or hTR associated with premature replicative senescence of the skin, e.g., the premature aging Werner syndrome, in Fanconi anaemia, and chronic dysplastic anaemia [37,112]. Positive effects of bone marrow-derived stem/progenitor cells for skin tissue engineering have been shown in several studies either alone or in combination with artificial skin grafts, thereby reducing the risk of graft rejection [106,108,113]. Ex vivo co-culture of human skin substitutes with circulating endothelial progenitor cells improved survival by formation of functional microvessels [107]. In a rat model transfection of hTERT into hair follicle stem cells by coating polyethylenimine DNA complexes on the skin surface stimulated hair growth [114], presumably by both telomere elongation and/or modulating Wnt/β-catenin signaling. Recently, optimized three-dimensional culture conditions have been described with enhanced hTERT expression levels, proliferation, and multipotency of human dermal stem/progenitor cells [115].

*Telomerase upregulation in atherosclerosis:* Despite enormous research and identification of numerous risk factors are the exact causes of atherosclerosis incompletely understood and the exact pathomechanism remains unclear [4,116]. Experimental findings in cell culture and animals suggest that telomere shortening contribute to the pathogenesis of atherosclerosis at advanced age. Numerous findings in humans have shown that telomere shortening correlates with the degree of atherosclerosis in vivo [15]. Experimental data suggest that the activation of telomerase can delay and—at least in part—reverse the senescent phenotype [15,117,118]. The pathologically vicious circle between replicative aging and inflammation by atherosclerosis could be reversed by a telomerase-based therapy [119]. Matsushita et al. have shown that a stable hTERT expression in endothelial cells ensures a younger phenotype and induces improvement of endothelial nitric oxide synthase (eNOS) [120]. Telomerase and vascular endothelial growth (VEGF)-mediated angiogenesis potentially regulate the transcriptional expression of each other, suggesting a role of telomerase in regulating cellular processes other than telomere elongation, such as differentiation and angiogenesis [119]. Development of therapeutic approaches is still on an experimental level due to fear of cancer-promoting side-effects on systemic use.

*Telomerase upregulation in psychiatric disorders:* Based on experimental and preliminary clinical data it was hypothesized that the mode of action of many psychopharmacological drugs (e.g., antidepressants, lithium, and antipsychotics) is, at least in part, mediated by their influence on telomerase activity. A close correlation between stress-dependent telomerase activity and depression-like behaviors has been shown in mice [121]. Fluoxetine reversed clinical symptoms and increased hippocampal telomerase activity in parallel, raising the possibility that drug effects might be mediated by telomerase-dependent neurogenesis [121]. Smaller pilot studies suggested a close correlation between telomerase activity, clinical symptoms and response to antidepressants [122,123]. Likewise, lithium increased hippocampal telomerase activity in a rat model of depression, accompanied by telomere elongation and reducing clinical symptoms [124]. In patients with bipolar disorder, telomere length correlated with duration of therapy [125]. Antipsychotic drugs may also have some positive influence on telomere length [126]. The modulation of intracellular Wnt/β-catenin or PI3K/Akt signaling pathways, the interaction with brain-derived neurotrophic factor and 5-HT, and antioxidant properties could represent possible mechanisms by which psychopharmacological drugs could modulate telomerase activity [127]. These pathways are functionally-relevant downstream drug effects and are also activation pathways for telomerase, suggesting a potential (yet unproven) mechanism by which these drugs may mediate neurogenesis via telomerae activation [38,128].

## 3. Telomerase as a Target for Cancer Treatment

There are several approaches for a telomerase-based gene therapy in the treatment of cancer. Cancer cells have high telomerase activity compared to most other cells [129]. The restriction of hTERT is a potential therapeutic option because telomerase complex components are up regulated in most tumor cells. Moreover, telomerase is a good target for cancer therapy because most somatic cells have no or only low level telomerase activities. Thus, the selective inactivation of telomerase expression in cancer cells does not influence most healthy cells [96]. Different therapeutic approaches for telomerase-based treatment of cancer have been developed or are under investigation [96,130].

*(1). Oligonucleotide inhibitors*. Antisense oligonucleotides and chemically-modified nucleic acids have been shown to inhibit telomerase and to induce telomere shortening [131,132,133,134,135] associated with subsequent onset of senescence and/or apoptosis in cell cultures [134,135,136,137]. These inhibitors act directly or indirectly (by inducing apoptosis). Targets include the RNA template, hTERT protein, and associated proteins. For example, the thio-phosphoramidate oligonucleotide inhibitor imetelstat (by Geron Corporation, Menlo Park, CA, USA) targets the RNA template for hTERT by binding to the catalytic site of telomerase [138]. Imetelstat (GRN163L) was successfully tested for glioblastoma tumors [139]. This cancer type ensures that there is sufficient time to permit tumor growth and erosion of telomeres to critical levels that trigger cellular senescence. Clinical phase II studies are planned for breast and lung cancers. Significant side-effects were not observed and possible combinatory therapies with well-established regimes for myeloproliferative neoplasms and acute myeloid leukemia are under investigation [96].

*(2). Small-molecule telomerase inhibitors*. Small-molecule telomerase inhibitors have been identified in screens of chemical libraries or were synthesized based on the structure of natural telomerase inhibitors such as epigallocatechin-3-gallate (EGCG) [140,141,142,143,144]. Moreover, various targets with overlapping functions have been proposed such as the PI3K-Akt-mTOR pathway [145], which is often dysregulated in cancer. The mTOR inhibitor rapamycin was shown to inhibit telomerase activity [146,147,148] and to counteract carcinogenesis.

*(3). Immunotherapeutic approaches*. The active site of telomerase in cancer cells is a possible target to develop vaccines [149]. Adoptive cell therapy, with the use of high-avidity T lymphocytes reactive against telomerase, has successfully been used in adenocarcinoma mouse prostate mice, which develop androgen-independent prostate cancer [150]. Despite marked temporary autoimmune depletion of B cells as side effect this therapy was not associated with significant immunoglobulin decreases or infections. At least 23 clinical studies, summarized in [151] have investigated hTERT immunotherapy as anticancer strategy in melanoma, acute myeloid leukemia, glioblastoma, prostate, renal, pancreatic, hepatocellular, and non-small-cell lung cancer: 18 phase I/I-II studies [152,153,154,155,156,157,158,159,160,161,162,163,164,165,166,167,168], four phase II studies [169,170,171,172], and one phase III trial [173] with pancreatic cancer patients and GV1001. Median survival ranged from 88 to 450 days in non-responders and from 216 to >600 days in responders [157,158,163,166,170,172], with the pancreatic cancer patients showing best survival rates so far. In a phase I trial an hTERT-derived peptide was used as a vaccine in hepatocellular carcinoma patients, with the majority of patients showing recurrence up to 24 weeks after vaccination [174].

Possible reasons for the limited success in many studies are the development of self-tolerance, the limited size of the precursor T-cell repertoire, negative effects of immunosuppressive tumor microenvironment on T cells, and interindividual differences. Various improvements have been proposed for future studies. These include (i) stimulation of cooperation between CD8+ and CD4+ T cells, by immunization with both MHC class I and class II hTERT peptides, in order to expand the pool of persisting memory CD8+ T cells; (ii) limiting the development of immune-tolerance, by immunization with low affinity (mutant) MHC I hTERT peptides, to increase the efficacy of vaccination; (iii) limiting the development of immune-tolerance by parallel immunization with peptides derived from non-self antigens; and (iv) development of personalized approaches with a focus on patients with early stage diseases to avoid negative effects of immunosuppressive cancer microenvironments [151].

*(4). Telomerase-directed gene therapy*. The promoters for telomerase in cancer cells are targets for a tumor specific gene therapy that selectively kills cancer cells and leaves normal cells unharmed by expressing high concentrations of a therapeutic protein only in cancer cells. Adenoviruses are developed that (by use of the hTERT promoter) selectively replicate in cancer cells and, subsequently, kill the cancer cells [175]. Both cytotoxic gene therapy and oncolytic virotherapy approaches have been used to kill cells expressing telomerase and not killing healthy cells [176].

*(5). Phytochemicals*. A wide variety of chemical compounds that occur naturally in plants, or phytochemicals, have been suggested to inhibit telomerase activity in various cancers, summarized in [177]. The substances include allicin, an organophosphate derived from garlic [178]; curcumin, a phenol present in turmeric [178,179,180,181,182,183,184,185]; the flavonolignan silbinin; an organosulfur derived from cruciferous vegetables; epigallocatechin gallate (EGCG), a catechin in green tea [186]. Curcumin [181], genistein [187], EGCG [188], and sulforaphane [189] were tested in breast cancer cells and the non-malignant breast cell line. The mode of action is only partially known and encompasses inhibition of translocation of hTERT to the nucleus [179]; dissociation of Hsp-90 co-chaperone from hTERT [183]; and a decrease of hTERT expression or activity [180,181,182,190].

Despite extensive studies during prior years on the development of telomerase vaccines, telomerase inhibitors, and telomerase promoter-driven cell killing in oncology, only one therapeutic vaccine went all the way to the clinic (GV1001), and only one telomerase antagonist (imetelstat, GRN163L) is in late preclinical studies. However, numerous drugs with various other targets have been identified with additional off-target effects on telomerase activity. These include substances which act via downregulation of hTERT gene transcription: the tyrosine kinase inhibitors dasatinib, imatinib, gefitinib, and nilotinib [191,192,193]; the ubiquitin/proteasome pathway inhibitor bortezomib; the cytotoxic drugs 5-azacytidine [194], arsenic trioxide [195] and temozolomide [196]; the chemosensitizer suramin [197]; the non-steroidal anti-inflammatory drugs aspirin [198], indomethacin [198], and celecoxib [199]; the peroxisome proliferator-activated receptor (PPAR) activator troglitazone [200]; the histone deacetylase inhibitors romidepsin [201] and vorinostat [202]; and the mTOR pathway inhibitor rapamycin [203]. The DNA topoisomerase I inhibitor beta-lapachone [204] and the DNA crosslinker cisplatin [205] act via downregulation of hTR gene transcription. The circadian rhythm hormone melatonin downregulates both hTERT and hTR on transcriptional level [206]. Other substances inhibit telomerase activity by unknown mechanisms: perifosine [207], nimesulide [208], auranofin [209], pyrimethamine [210], azidothymidine [211], octreotide [212] and ofloxacin [213]. Quinacrine, bortezomib, etoposide, and doxorubicin directly target the telomere structure proteins TRF1, POT1, shelterin, and TNKS1 [214,215,216,217].

Various drugs proposed for skin cancer therapy, including tyrosine kinase and Wnt/β-catenin signaling inhibitors, also have inhibitory effects on telomerase [218,219,220,221,222,223]. This is not unexpected because telomerase enzyme activity can be post-transcriptionally regulated by the kinases c-Abl, protein kinase C, ERK1/2, and Akt. [224,225,226,227,228,229,230,231,232,233,234].

Blockade of the epidermal growth factor receptor might be effective in inhibiting telomerase activity of squamous cell carcinomas, which may result in suppression of tumor growth [235]. The recent finding of a germline mutation in the promoter of hTERT in a melanoma-prone family further suggests the importance of telomerase as an important target in skin cancer therapy [236]. Resveratrol, by contrast is, at least in mouse models, a potent chemopreventive agent against melanoma [237,238] but rather increases telomerase activity.

## 4. Advantages, Pitfalls, and Outlook

There are many advantages for using telomerase as an anti-cancer target. First, it is an essential and specific component for most cancer cells [129,138] and more widely expressed than any other tumor marker. Approximately 90% of all human cancers have elevated telomerase levels relative to normal cells. Second, telomerase is the most efficient mechanism for replicative immortality with only one (less robust) compensatory mechanism (ALT), which limits the risk for development of resistance to telomerase-based therapies. Third, the very low expression of telomerase in normal tissues, together with the longer telomeres in normal stem cells versus cancer cells, provides at least some degree of specificity with low risk of toxicity in normal cells [239], and limited risk in stem cells, provided that telomerase inhibition is limited in time.

There are also various pitfalls for a use of telomerase as a therapeutic target in cancer treatment. First, the anti-proliferative effects of telomerase inhibition are induced in cells with short telomeres only, which requires some time of tumor growth until the drug can be effective. Second, telomerase inhibition may elicit negative effects in highly proliferative cells which need telomerase for survival, namely, stem cells, etc. [240]. Despite shorter telomeres in cancer cells, these two points may restrict the therapeutic option for a narrow telomere length window. Moreover, it has been shown that stress induces a shift of telomerase from the nucleus to mitochondria, suggesting a telomere-independent physiological function and possible risks in long term inhibition of telomerase [16]. There are also caveats to the therapeutic strategy of senescence induction, per se. On one hand, both telomere-driven replicative senescence and stress-inducible senescence are tumor-suppressive [241,242,243,244,245,246,247]. On the other hand, senescence can be reversed by a process called crisis and then has pro-tumorigenic paracrine effects. By induction of telomere dysfunction and attrition chromosomal instability occurs and may result in activation of oncogenes and/or silencing of tumor suppressor genes, which may counteract the original therapeutic intention and cooperate to promote malignant transformation and drug resistance [248,249,250,251,252]. Finally, the currently available drugs and substances are often unspecific and have a wide variety of actions and different cellular targets that may counteract replicative immortality but may also exacerbate other cancer hallmarks such as chromosomal instability. Some studies are promising but also suggest that for complete eradication of all subpopulations of cancer cells a simultaneous targeting of several mechanisms will be needed. It is unlikely that a single target will provide lasting remission.

Approximately 5%–10% of cancer cells maintain their telomeres by ALT [28]. This process is only partially understood but may offer new therapeutic options by modulating the involved factors such as the shelterin complex or the telomere sequences themselves to induce telomere deprotection.

There are also a number of pitfalls in the treatment with telomerase-activating procedures or substances. Immortality is not intrinsically essential for malignancy [253]. However, an extended lifespan may accumulate rare genetic and epigenetic aberrations that can contribute to malignant transformation. Constitutive telomerase expression in mice increased tissue fitness and delay of aging at the expense of slightly increased cancer incidence [254,255,256]. In mice with cancer resistant backgrounds (by increased expression of tumor suppressors p16, Arf, and p53) transgenic telomerase expression extends lifespan by 43% [254]. Interestingly, the cancer-promoting activity in transgenic mouse models is not observed when telomerase is re-activated later in life.

In this context, a recently described methodological advance is of interest. Bernardes de Jesus et al. have designed a potential therapeutic approach in which telomerase is induced temporarily and selectively in old cells without promoting cancer growth. The special feature of recombinant adeno-associated virus (rAAV) vector is its ‘mild’ integration into the host genome. By use of rAAV vectors, which expressed the catalytic subunit of mouse telomerase (mTERT) it was integrated into the host genome at very low rates but, in mice, did not induce cancer growth. A possible explanation is the loss of the vector in rapidly proliferating cells such as cancer cells. Thus, this method might be used to treat age-related diseases, such as atherosclerosis or diabetes [257]. In short-lived organisms, such as mice, this strategy seems to be an excellent approach for a telomerase-based gene therapy. For long-lived organisms it is currently unknown if cancer can be promoted by rare integration events of constitutively-overexpressed hTERT [257].

## Abbreviations

The following abbreviations are used in this manuscript:

ALT	alternative lengthening of telomeres
CRP	C-reactive protein
hTERT	human telomerase reverse transcriptase
hTR	human telomerase RNA
IL-6	interleukine-6
LTL	leucocyte telomere length
mTERT	mouse telomerase reverse transcriptase
rAAV	recombinant adeno-associated virus
ROS	reactive oxygen species
TERC	telomerase RNA component
TERT	telomerase reverse transcriptase
VEGF	vascular endothelial growth

## References

[1] Walter M. (2009). Interrelationships among HDL metabolism, aging, and atherosclerosis. Arterioscler Thromb. Vasc. Biol..

[2] Fyhrquist F., Saijonmaa O., Strandberg T. (2013). The roles of senescence and telomere shortening in cardiovascular disease. Nat. Rev. Cardiol..

[3] To-Miles F.Y.L., Backman C.L. (2016). What telomeres say about activity and health: A rapid review. Can. J. Occup. Ther..

[4] Zhu H., Blecher M., van der Harst P. (2011). Healthy aging and disease: Role for telomere biology?. Clin. Sci..

[5] Boccardi V., Herbig U. (2012). Telomerase gene therapy: A novel approach to combat aging. EMBO Mol. Med..

[6] Xin H., Liu D., Songyang Z. (2008). The telosome/shelterin complex and its functions. Genome Biol..

[7] De Lange T. (2005). Shelterin: The protein complex that shapes and safeguards human telomeres. Genes Dev..

[8] Artandi S.E., DePinho R.A. (2010). Telomeres and telomerase in cancer. Carcinogenesis.

[9] Sarin K.Y., Cheung P., Gilison D., Lee E., Tennen R.I., Wang E., Artandi M.K., Oro A.E., Artandi S.E. (2005). Conditional telomerase induction causes proliferation of hair follicle stem cells. Nature.

[10] Baker D.J., Wijshake T., Tchkonia T., LeBrasseur N.K., Childs B.G., van de Sluis B., Kirkland J.L., van Deursen J.M. (2001). Clearance of p16Ink4apositive senescent cells delays ageing associated disorders. Nature.

[11] Stanley S.E., Armanios M. (2015). The short and long telomere syndromes: Paired paradigms for molecular medicine. Curr. Opin. Gen. Dev..

[12] Park J.I., Venteicher A.S., Hong J.Y., Choi J., Jun S., Shkreli M., Chang W., Meng Z., Cheung P., Ji H. (2009). Telomerase modulates Wnt signalling by association with target gene chromatin. Nature.

[13] Rao T.P., Kuhl M. (2010). An updated overview on Wnt signaling pathways: A prelude for more. Circ. Res..

[14] Von Zglinicki T. (2001). Oxidative stress shortens telomeres. Trends Biochem. Sci..

[15] Nazari-Shafti T.Z., Cooke J.P. (2015). Telomerase therapy to reserve cardiovascular senescence. Methodist Debakey Cardiovasc. J..

[16] Babizhayev M.A., Yegorov Y.E. (2015). Tissue formation and tissue engineering through host cell recruitment or a potential injectable cell-based biocomposite with replicative potential: Molecular mechanisms controlling cellular senescence and the involvement of controlled transient telomerase activation therapies. J. Biomed. Mater. Res. Part A.

[17] Xu D., Erickson S., Szeps M., Gruber A., Sangfelt O., Einhorn S., Pisa P., Grandér D. (2000). Interferon α down-regulates telomerase reverse transcriptase and telomerase activity in human malignant and nonmalignant hematopoietic cells. Blood.

[18] Aviv A., Valdes A., Gardner J.P., Swaminathan R., Kimura M., Spector T.D. (2006). Menopause modifies the association of leukocyte telomere length with insulin resistance and inflammation. J. Clin. Endocrinol. Metab..

[19] Carrero J.J., Stenvinkel P., Fellstrom B., Qureshi A.R., Lamb K., Heimbürger O., Bárány P., Radhakrishnan K., Lindholm B., Soveri I. (2008). Telomere attrition is associated with inflammation, low fetuin—a levels and high mortality in prevalent haemodialysis patients. J. Intern. Med..

[20] Deng W., Cheung S.T., Tsao S.W., Wang X.M., Tiwari A.F. (2016). Telomerase activity and its association with psychological stress, mental disorders, lifestyle factors and interventions: A systematic review. Psychoneuroendocrinology.

[21] Oliveira B.S., Zunzunegui M.V., Quinlan J., Fahmi H., Tu M.T., Guerra R.O. (2016). Systematic review of the association between chronic social stress and telomere length: A life course perspective. Ageing Res. Rev..

[22] Nawrot T.S., Staessen J.A., Holvoet P., Struijker-Boudier H.A., Schiffers P., van Bortel L.M., Fagard R.H., Gardner J.P., Kimura M., Aviv A. (2010). Telomere length and its associations with oxidized-LDL, carotid artery distensibility and smoking. Front. Biosci..

[23] Yaswen P., MacKenzieb K.L., Keithc W.N., Hentosh P., Rodier F., Zhu J., Firestone G.L., Matheu A., Carnero A., Bilsland A. (2015). Therapeutic targeting of replicative immortality. Sem. Cancer Biol..

[24] Jacobs J.J.L., de Lange T. (2004). Significant Role for p16^INK4a^ in p53-Independent Telomere-Directed Senescence. Curr. Biol..

[25] Jacobs J.J.L. (2013). Loss of Telomere Protection: Consequences and Opportunities. Front. Oncol..

[26] Chang S., Khoo C.M., Naylor M.L., Maser R.S., DePinho R.A. (2003). Telomere-based crisis: Functional differences between telomerase activation and ALT in tumor progression. Genes Dev..

[27] Hu J., Hwang S.S., Liesa M., Gan B., Sahin E., Jaskelioff M., Ding Z., Ying H., Boutin A.T., Zhang H. (2012). Antitelomerase Therapy Provokes ALT and Mitochondrial Adaptive Mechanisms in Cancer. Cell.

[28] O’Sullivan R., Almouzni G. (2014). Assembly of telomeric chromatin to create ALTernative endings. Trends Cell Biol..

[29] Zhang A., Zheng C., Lindvall C., Hou M., Ekedahl J., Lewensohn R., Yan Z., Yang X., Henriksson M., Blennow E. (2000). Frequent Amplification of the Telomerase Reverse Transcriptase Gene in Human Tumors. Cancer Res..

[30] Zhang A., Zheng C., Hou M., Lindvall C., Wallin K.L., Angström T., Yang X., Hellström A.C., Blennow E., Björkholm M. (2002). Amplification of the Telomerase Reverse Transcriptase (hTERT) Gene in Cervical Carcinomas. Genes Chromos. Cancer.

[31] Takuma Y., Nouso K., Kobayashi Y., Nakamura S., Tanaka H., Matsumoto E., Fujikawa T., Suzuki M., Hanafusa T., Shiratori Y. (2004). Telomerase reverse transcriptase gene amplification in hepatocellular carcinoma. J. Gastroenterol. Hepatol..

[32] Rousseau P., Autexier C. (2016). Telomere biology: Rationale for diagnostics and therapeutics in cancer. RNA Biol..

[33] Teralı K., Yilmazer A. (2016). New surprises from an old favourite: The emergence of telomerase as a key player in the regulation of cancer stemness. Biochimie.

[34] Soder A.I., Hoare S.F., Muir S., Going J.J., Parkinson E.K., Keith W.N. (1997). Amplification, increased dosage and in situ expression of the telomerase RNA gene in human cancer. Oncogene.

[35] Shay J.W., Wright W.E. (2007). Hallmarks of telomeres in ageing research. J. Pathol..

[36] Sahin E., Depinho R.A. (2010). Linking functional decline of telomeres, mitochondria and stem cells during ageing. Nature.

[37] Shay J.W., Wright W.E. (2005). Use of telomerase to create bioengineered tissues. Ann. N. Y. Acad. Sci..

[38] Jaskelioff M., Muller F.L., Paik J.H., Thomas E., Jiang S., Adams A.C., Sahin E., Kost-Alimova M., Protopopov A., Cadiñanos J. (2011). Telomerase reactivation reverses tissue degeneration in aged telomerase-deficient mice. Nature.

[39] Allsopp R.C., Weissman I.L. (2002). Replicative senescence of hematiopoietic stem cells during serial transplantation: Does telomere shortening play a role?. Oncogene.

[40] Pendino F., Tarkanyi I., Dudognon C., Hillion J., Lanotte M., Aradi J., Segal-Bendirdjian S. (2006). Telomeres and telomerase: Pharmacological targets for new anticancer strategies?. Curr. Cancer Drug Targets.

[41] Atkinson S.P., Hoare S.F., Glasspool R.M., Keith W.N. (2005). Lack of telomerase gene expression in alternative lengthening of telomere cells is associated with chromatin remodeling of the hTR and hTERT gene promoters. Cancer Res..

[42] Serenicki N., Hoare S.F., Kassem M., Atkinson S.P., Keith W.N. (2006). Telomerase promoter reprogramming and interaction with general transcription factors in the human mesenchymal stem cell. Regen. Med..

[43] Doshida M., Ohmichi M., Tsutsumi S., Kawagoe J., Takahashi T., Du B., Mori-Abe A., Ohte T., Saitoh-Sekiguchi M., Takahashi K. (2006). Raloxifene increases proliferation and up-regulates telomerase activity in human umbilical vein endothelial cells. J. Biol. Chem..

[44] Liu K., Hodes R.J., Weng N. (2001). Cutting edge: Telomerase activation in human T lymphocytes does not require increase in telomerase reverse transcriptase (hTERT) protein but is associated with hTERT phosphorylation and nuclear translocation. J. Immunol..

[45] Tarkanyi I., Aradi J. (2008). Pharmacological intervention strategies for affecting telomerase activity: Future prospects to treat cancer and degenerative disease. Biochemie.

[46] Stewart S.A. (2002). Multiple levels of telomerase regulation. Mol. Interv..

[47] Sharma N.K., Reyes A., Green P., Caron M.J., Bonini M.G., Gordon D.M., Holt I.J., Santos J.H. (2012). Human telomerase acts as a hTR-independent reverse transcriptase in mitochondria. Nucleic Acids Res..

[48] Bodnar A.G., Ouellette M., Frolkis M., Holt S.E., Chiu C.P., Morin G.B., Harley C.B., Shay J.W., Lichtsteiner S., Wright W.E. (1998). Extension of life-span by introduction of telomerase into normal human cells. Science.

[49] Counter C.M., Meyerson M., Eaton E.N., Ellisen L.W., Caddle S.D., Haber D.A., Weinberg R.A. (1998). Telomerase activity is restored in human cells by ectopic expression of hTERT (hEST2), the catalytic subunit of telomerase. Oncogene.

[50] Vaziri H., Benchimol S. (1998). Reconstitution of telomerase activity in normal human cells leads to elongation of telomeres and extended replicative life span. Curr. Biol..

[51] Jiang X.R., Jimenez G., Chang E., Frolkis M., Kusler B., Sage M., Beeche M., Bodnar A.G., Wahl G.M., Tlsty T.D. (1999). Telomerase expression in human somatic cells does not induce changes associated with a transformed phenotype. Nat. Genet..

[52] Morales C.P., Holt S.E., Ouellette M., Kaur K.J., Yan Y., Wilson K.S., White M.A., Wright W.E., Shay J.W. (1999). Absence of cancer-associated changes in human fibroblasts immortalized with telomerase. Nat. Genet..

[53] Funk W.D., Wang C.K., Shelton D.N., Harley C.B., Pagon G.D., Hoeffler W.K. (2000). Telomerase expression restores dermal integrity to in vitro-aged fibroblasts in a reconstituted skin model. Exp. Cell Res..

[54] Wyllie F.S., Jones C.J., Skinner J.W., Haughton M.F., Wallis C., Wynford-Thomas D., Faragher R.G., Kipling D. (2000). Telomerase prevents the accelerated cell ageing of Werner syndrome fibroblasts. Nat. Genet..

[55] Harada H., Nakagawa H., Takaoka M., Lee J., Herlyn M., Diehl J.A., Rustgi A.K. (2008). Cleavage of MCM2 licensing protein fosters senescence in human keratinocytes. Cell Cycle.

[56] McKee J.A., Banik S.S., Boyer M.J., Hamad N.M., Lawson J.H., Niklason L.E., Counter C.M. (2003). Human arteries engineered in vitro. EMBO Rep..

[57] Oh H., Taffet G.E., Youker K.A., Entman M.L., Overbeek P.A., Michael L.H., Schneider M.D. (2001). Telomerase reverse transcriptase promotes cardiac muscle cell proliferation, hypertrophy, and survival. Proc. Natl. Acad. Sci. USA.

[58] Wootton M., Steeghs K., Watt D., Munro J., Gordon K., Ireland H., Morrison V., Behan W., Parkinson E.K. (2003). Telomerase alone extends the replicative life span of human skeletal muscle cells without compromising genomic stability. Hum. Gene Ther..

[59] Yang J., Chang E., Cherry A.M., Bangs C.D., Oei Y., Bodnar A., Bronstein A., Chiu C.P., Herron G.S. (1999). Human endothelial cell life extension by telomerase expression. J. Biol. Chem..

[60] Gu X., Zhang J., Brann D.W., Yu F.S. (2003). Brain and retinal vascular endothelial cells with extended life span established by ectopic expression of telomerase. Invest. Ophthalmol. Vis. Sci..

[61] Yang J., Nagavarapu U., Relloma K., Sjaastad M.D., Moss W.C., Passaniti A., Herron G.S. (2001). Telomerized human microvasculature is functional in vivo. Nat. Biotechnol..

[62] Condon J., Yin S., Mayhew B., Word R.A., Wright W.E., Shay J.W., Rainey W.E. (2002). Telomerase immortalization of human myometrial cells. Biol. Reprod..

[63] Shi S., Gronthos S., Chen S., Reddi A., Counter C.M., Robey P.G., Wang C.Y. (2002). Bone formation by human postnatal bone marrow stromal stem cells is enhanced by telomerase expression. Nat. Biotechnol..

[64] Simonsen J.L., Rosada C., Serakinci N., Justesen J., Stenderup K., Rattan S.I., Jensen T.G., Kassem M. (2002). Telomerase expression extends the proliferative life-span and maintains the osteogenic potential of human bone marrow stromal cells. Nat. Biotechnol..

[65] Gronthos S., Chen S., Wang C.Y., Robey P.G., Shi S. (2003). Telomerase accelerates osteogenesis of bone marrow stromal stem cells by upregulation of CBFA1, osterix, and osteocalcin. J. Bone Miner Res..

[66] Kawano Y., Kobune M., Yamaguchi M., Nakamura K., Ito Y., Sasaki K., Takahashi S., Nakamura T., Chiba H., Sato T. (2003). Ex vivo expansion of human umbilical cord hematopoietic progenitor cells using a coculture system with human telomerase catalytic subunit (hTERT)-transfected human stromal cells. Blood.

[67] Darimont C., Avanti O., Tromvoukis Y., Vautravers-Leone P., Kurihara N., Roodman G.D., Colgin L.M., Tullberg-Reinert H., Pfeifer A.M., Offord E.A. (2002). SV40 T antigen and telomerase are required to obtain immortalized human adult bone cells without loss of the differentiated phenotype. Cell Growth Differ..

[68] Yudoh K., Matsuno H., Nakazawa F., Katayama R., Kimura T. (2001). Reconstituting telomerase activity using the telomerase catalytic subunit prevents the telomere shorting and replicative senescence in human osteoblasts. J. Bone Miner Res..

[69] Yudoh K., Nishioka K. (2004). Telomerized presenescent osteoblasts prevent bone mass loss in vivo. Gene Ther..

[70] Hao J., Narayanan K., Ramachandran A., He G., Almushayt A., Evans C., George A. (2002). Odontoblast cells immortalized by telomerase produce mineralized dentin-like tissue both in vitro and in vivo. J. Biol. Chem..

[71] Luiten R.M., Pene J., Yssel H., Spits H. (2003). Ectopic hTERT expression extends the life span of human CD4 helper and regulatory T-cell clones and confers resistance to oxidative stress-induced apoptosis. Blood.

[72] Kobune M., Kawano Y., Ito Y., Chiba H., Nakamura K., Tsuda H., Sasaki K., Dehari H., Uchida H., Honmou O. (2003). Telomerized human multipotent mesenchymal cells can differentiate into hematopoietic and cobblestone area-supporting cells. Exp. Hematol..

[73] Di Donna S., Mamchaoui K., Cooper R.N., Seigneurin-Venin S., Tremblay J., Butler-Browne G.S., Mouly V. (2003). Telomerase can extend the proliferative capacity of human myoblasts, but does not lead to their immortalization. Mol. Cancer Res..

[74] Schnabl B., Choi Y.H., Olsen J.C., Hagedorn C.H., Brenner D.A. (2002). Immortal activated human hepatic stellate cells generated by ectopic telomerase expression. Lab. Invest..

[75] Watanabe T., Shibata N., Westerman K.A., Okitsu T., Allain J.E., Sakaguchi M., Totsugawa T., Maruyama M., Matsumura T., Noguchi H. (2003). Establishment of immortalized human hepatic stellate scavenger cells to develop bioartificial livers. Transplantation.

[76] Roy N.S., Nakano T., Keyoung H.M., Windrem M., Rashbaum W.K., Alonso M.L., Kang J., Peng W., Carpenter M.K., Lin J. (2004). Telomerase immortalization of neuronally restricted progenitor cells derived from the human fetal spinal cord. Nat. Biotechnol..

[77] Townsley D.M., Dumitriu B., Young N.S. (2014). Bone marrow failure and the telomeropathies. Blood.

[78] Shay J.W., Wright W.E. (2000). The use of telomerized cells for tissue engineering. Nat. Biotechnol..

[79] Ulaner G.A. (2004). Telomere Maintenance in Clinical Medicine. Am. J. Med..

[80] Bär C., Povedano J.M., Serrano R., Benitez-Buelga C., Popkes M., Formentini I., Bobadilla M., Bosch F., Blasco M.A. (2016). Telomerase gene therapy rescues telomere length, bone marrow aplasia, and survival in mice with aplastic anemia. Blood.

[81] Donati B., Valenti L. (2016). Telomeres, NAFLD and Chronic Liver Disease. Int. J. Mol. Sci..

[82] Calado R.T. (2014). Telomeres in lung diseases. Prog. Mol. Biol. Transl. Sci..

[83] Li J., Pei M. (2012). Cell senescence: A challenge in cartilage engineering and regeneration. Tissue Eng. Part B Rev..

[84] Babizhayev M.A., Yegorov Y.E. (2010). Telomere attrition in lens epithelial cells—A target for N-acetylcarnosine therapy. Front. Biosci..

[85] Weyand C.M., Fujii H., Shao L., Goronzy J.J. (2009). Rejuvenating the immune system in rheumatoid arthritis. Nat. Rev. Rheumatol..

[86] Thomas M., Yang L., Hornsby P.J. (2000). Formation of functional tissue from transplanted adrenocortical cells expressing telomerase reverse transcriptase. Nat. Biotechnol..

[87] Fauce S.R., Jamieson B.D., Chin A.C., Mitsuyasu R.T., Parish S.T., Ng H.L., Kitchen C.M., Yang O.O., Harley C.B., Effros R.B. (2008). Telomerase-based pharmacologic enhancement of antiviral function of human CD8+ T lymphocytes. J. Immunol..

[88] Dock J.N., Effros R.B. (2011). Role of CD8 T Cell Replicative Senescence in Human Aging and in HIV-mediated Immunosenescence. Aging Dis..

[89] Harley C.B., Liu W., Blasco M., Vera E., Andrews W.H., Briggs L.A., Raffaele J.M. (2011). A natural product telomerase activator as part of a health maintenance program. Rejuven. Res..

[90] Bernardes de Jesus B., Schneeberger K., Vera E., Tejera A., Harley C.B., Blasco M.A. (2011). The telomerase activator TA-65 elongates short telomeres and increases health span of adult/old mice without increasing cancer incidence. Aging Cell.

[91] Dow C.T., Harley C.B. (2016). Evaluation of an oral telomerase activator for early age-related macular degeneration—A pilot study. Clin. Ophthalmol..

[92] Harley C.B., Liu W., Flom P.L., Raffaele J.M. (2013). A natural product telomerase activator as part of a health maintenance program: Metabolic and cardiovascular response. Rejuven. Res..

[93] Salvador L., Singaravelu G., Harley C.B., Flom P., Suram A., Raffaele J.M. (2016). A Natural Product Telomerase Activator Lengthens Telomeres in Humans: A Randomized, Double Blind, and Placebo Controlled Study. Rejuven. Res..

[94] Pearce V.P., Sherrell J., Lou Z., Kopelovich L., Wright W.E., Shay J.W. (2008). Immortalization of epithelial progenitor cells mediated by resveratrol. Oncogene.

[95] Xia L., Wang X.X., Hu X.S., Guo X.G., Shang Y.P., Chen H.J., Zeng C.L., Zhang F.R., Chen J.Z. (2008). Resveratrol reduces endothelial progenitor cells senescence through augmentation of telomerase activity by Akt-dependent mechanisms. Br. J. Pharmacol..

[96] Sprouse A.A., Steding C.E., Herbert B.-S. (2012). Pharmaceutical regulation of telomerase and its clinical potential. J. Cell. Mol. Med..

[97] Eitan E., Tichon A., Gazit A., Gitler D., Slavin S., Priel E. (2012). Novel telomerase-increasing compound in mouse brain delays the onset of amyotrophic lateral sclerosis. EMBO Mol. Med..

[98] Wang F., Chang G., Geng X. (2014). NGF and TERT co-transfected BMSCs improve the restoration of cognitive impairment in vascular dementia rats. PLoS ONE.

[99] Haendeler J., Hoffman J., Diehl J.F., Vasa M., Spyridopoulos I., Zeiher A.M., Dimmeler S. (2004). Antioxidants inhibit nuclear export of telomerase reverse transcriptase and delay replicative senescence of endothelial cells. Circ. Res..

[100] Tanaka Y., Moritoh Y., Miva N. (2007). Age-dependent telomere-shortening is repressed by phosphorylated alpha-tocopherol together with cellular longevity and intracellular oxidative-stress reduction in human brain microvascular endotheliocytes. J. Cell Biochem..

[101] Passos J.F., Saretzki G., Ahmed S., Nelson G., Richter T., Peters H., Wappler I., Birket M.J., Harold G., Schaeuble K. (2007). Mitochondrial dysfunction accounts for the stochastic heterogeneity in telomere-dependent senescence. PLoS Biol..

[102] Brouilette S.W., Moore J.S., McMahon A.D., Thompson J.R., Ford I., Shepherd J., Packard C.J., Samani N.J. (2007). Telomere length, risk of coronary heart disease, and statin treatment in the West of Scotland Primary Prevention Study: A nested case-control study. Lancet.

[103] Spyridopoulos I., Haendeler J., Urbich C., Brummendorf T.H., Oh H., Schneider M.D., Zeiher A.M., Dimmeler S. (2004). Statins enhance migratory capacity by upregulation of the telomere repeat binding factor TRF2 in endothelial progenitor cells. Circulation.

[104] Dong X.X., Hui Y.J., Xiang W.X., Rong Z.F., Jian S., Zhu C.J. (2007). Ginkgo Biloba extract reduces endothelial progenitor-cell senescence trough augmentation of telomerase activity. J. Cardiovasc. Pharmacol..

[105] Mimeault M., Batra S.K. (2006). Recent advances on the significance of stem cells in tissue regeneration and cancer therapies. Stem Cells.

[106] Yoshikawa T., Mitsuno H., Nonaka I., Sen Y., Kawanishi K., Inada Y., Takakura Y., Okuchi K., Nonomura A. (2008). Wound therapy by marrow mesenchymal cell transplantation. Plast. Reconstr. Surg..

[107] Kung E.F., Wang F., Schechner J.S. (2008). In vivo perfusion of human skin substitutes with microvessels formed by adult circulating endothelial progenitor cells. Dermatol. Surg..

[108] Zhang C.P., Fu X.B. (2008). Therapeutic potential of stem cells in skin repair and regeneration. Chin. J. Traumatol..

[109] Park B.S., Jang K.A., Sung J.H., Park J.S., Kwon Y.H., Kim K.J., Kim W.S. (2008). Adipose-derived stem cells and their secretory factors as a promising therapy for skin aging. Dermatol. Surg..

[110] Branski L.K., Gauglitz G.G., Herndon D.N., Jeschke M.G. (2008). A review of gene and stem cell therapy in cutaneous wound healing. Burns.

[111] Kim D.S., Cho H.J., Yang S.K., Shin J.W., Huh C.H., Park K.C. (2009). IGFBP-2 Contributes to the proliferation of less proliferative cells in forming skin equivalents. Tissue Eng. Part A.

[112] Siegl-Cachedenier I., Flores I., Klatt P., Blasco M.A. (2007). Telomerase reverses epidermal hair follicle stem cell defects and loss of longterm survival associated with critically short telomeres. J. Cell Biol..

[113] Wu Y., Wang J., Scott P.G., Tredget E.E. (2007). Bone marrow-derived stem cells in wound healing: A review. Wound Repair Regen..

[114] Jan H.M., Wei M.F., Peng C.L., Lin S.J., Lai P.S., Shieh M.J. (2012). The use of polyethylenimine-DNA to topically deliver hTERT to promote hair growth. Gene Ther..

[115] Shim J.H., Lee T.R., Shin D.W. (2013). Novel in vitro culture condition improves the stemness of human dermal stem/progenitor cells. Mol. Cells.

[116] Neuner B., Lenfers A., Kelsch R., Jäger K., Brüggmann N., van der Harst P., Walter M. (2015). Telomere length is not related to established cardiovascular risk factors but does correlate with red and white blood cell counts in a German blood donor population. PLoS ONE.

[117] Walter M., Forsyth N.R., Wright W., Shay J.W., Roth M.G. (2004). The establishment of telomerase-immortalized Tangier disease cell lines indicates the existence of an apolipoprotein A-I-inducible but ABCA1-independent cholesterol efflux pathway. J. Biol. Chem..

[118] Kannenberg F., Gorzelniak K., Jäger K., Fobker M., Rust S., Repa J., Roth M., Björkhem I., Walter M. (2013). Characterization of cholesterol homeostasis in telomerase-immortalized Tangier disease fibroblasts reveals marked phenotype variability. J. Biol. Chem..

[119] Hartwig F.P., Nedel F., Collares T.V., Tarquinio S.B., Nör J.E., Demarco F.F. (2012). Telomeres and tissue engineering: The potential roles of TERT in VEGF-mediated angiogenesis. Stem Cell Rev..

[120] Matsushita H., Chang E., Glassford A.J., Cooke J.P., Chiu C.P., Tsao P.S. (2001). eNOS Activity Is Reduced in Senescent Human Endothelial Cells Preservation by hTERT Immortalization. Circ. Res..

[121] Zhou Q.G., Hu Y., Wu D.L., Zhu L.J., Chen C., Jin X., Luo C.X., Wu H.Y., Zhang J., Zhu D.Y. (2011). Hippocampal telomerase is involved in the modulation of depressive behaviors. J. Neurosci..

[122] Wolkowitz O.M., Mellon S.H., Epel E.S., Lin J., Reus V.I., Rosser R., Burke H., Compagnone M., Nelson J.C., Dhabhar F.S. (2012). Resting leukocyte telomerase activity is elevated in major depression and predicts treatment response. Mol. Psychiatry.

[123] Simon N.M., Walton Z.E., Bui E., Prescott J., Hoge E., Keshaviah A., Schwarz N., Dryman T., Ojserkis R.A., Kovachy B. (2015). Telomere length and telomerase in a well-characterized sample of individuals with major depressive disorder compared to controls. Psychoneuroendocrinology.

[124] Wei Y.B., Backlund L., Wegener G., Mathé A.A., Lavebratt C. (2015). Telomerase dysregulation in the hippocampus of a rat model of depression. Normalization by lithium. Int. J. Neuropsychopharmacol..

[125] Martinsson L., Wei Y., Xu D., Melas P.A., Mathé A.A., Schalling M., Lavebratt C., Backlund L. (2013). Long-term lithium treatment in bipolar disorder is associated with longer leukocyte telomeres. Transl. Psychiatry.

[126] Yu W.Y., Chang H.W., Lin C.H., Cho C.L. (2008). Short telomeres in patients with chronic schizophrenia who show a poor response to treatment. J. Psychiatry Neurosci..

[127] Bersani F.S., Lindqvist D., Mellon S.H., Penninx B.W., Verhoeven J.E., Révész D., Reus V.I., Wolkowitz O.M. (2015). Telomerase activation as a possible mechanism of action for psychopharmacological interventions. Drug Discov. Today.

[128] Duman R.S., Malberg J., Nakagawa S. (2001). Regulation of adult neurogenesis by psychotropic drugs and stress. J. Pharmacol. Exp. Ther..

[129] Kim N.W., Piatyszek M.A., Prowse K.R., Harley C.B., West M.D., Ho P.L., Coviello G.M., Wright W.E., Weinrich S.L., Shay J.W. (1994). Specific association of human telomerase activity with immortal cells and cancer. Science.

[130] Podlevsky J.D., Chen J.J. (2012). It all comes together at the ends: Telomerase structure, function, and biogenesis. Mutat. Res..

[131] Feng J., Funk W.D., Wang S.-S., Weinrich S.L., Avilion A.A., Chiu C.P., Adams R.R., Chang E., Allsopp R.C., Yu J. (1995). The RNA component of human telomerase. Science.

[132] Norton J.C., Piatyszek M.A., Wright W.E., Shay J.W., Corey D.R. (1996). Inhibition of human telomerase activity by peptide nucleic acids. Nat. Biotechnol..

[133] Kondo S., Tanaka Y., Kondo Y., Hitomi M., Barnett G.H., Ishizaka Y., Liu J., Haqqi T., Nishiyama A., Villeponteau B. (1988). Antisense telomerase treatment: Induction of two distinct pathways, apoptosis and differentiation. FASEB J..

[134] Pitts A.E., Corey D.R. (1988). Inhibition of human telomerase by 2’-O-methyl-RNA. Proc. Natl. Acad. Sci. USA.

[135] Herbert B., Pitts A.E., Baker S.I., Hamilton S.E., Wright W.E., Shay J.W., Corey D.R. (1999). Inhibition of human telomerase in immortal human cells leads to progressive telomere shortening and cell death. Proc. Natl. Acad. Sci. USA.

[136] Corey D.R. (2002). Telomerase inhibition, oligonucleotides, and clinical trials. Oncogene.

[137] Asai A., Oshima Y., Yamamoto Y., Uochi T.A., Kusaka H., Akinaga S., Yamashita Y., Pongracz K., Pruzan R., Wunder E. (2003). A novel telomerase template antagonist (GRN163) as a potential anticancer agent. Cancer Res..

[138] Harley C.B. (2008). Telomerase and cancer therapeutics. Nat. Rev. Cancer.

[139] Marian C.O., Cho S.K., McEllin B.M., Maher E.A., Hatanpaa K.J., Madden C.J., Mickey B.E., Wright W.E., Shay J.W., Bachoo R.M. (2010). The telomerase antagonist, imetelstat, efficiently targets glioblastoma tumor-initiating cells leading to decreased proliferation and tumor growth. Clin. Cancer Res..

[140] Naasani I., Seimiya H., Yamori T., Tsuruo T. (1999). FJ5002: A potent telomerase inhibitor identified by exploiting the disease-oriented screening program with COMPARE analysis. Cancer Res..

[141] Hayakawa N., Nozawa K., Ogawa A., Kato N., Yoshida K., Akamatsu K.I., Tsuchiya M., Nagasaka A., Yoshida S. (1999). Isothiazolone derivatives selectively inhibit telomerase from human and rat cancer cells in vitro. Biochemistry.

[142] Damm K., Hemmann U., Garin-Chesa P., Hauel N., Kauffmann I., Priepke H., Niestroj C., Daiber C., Enenkel B., Guilliard B. (2001). A highly selective telomerase inhibitor limiting human cancer cell proliferation. EMBO J..

[143] Kleideiter E., Piotrowska K., Klotz U. (2007). Screening of telomerase inhibitors. Methods Mol. Biol..

[144] Wong L.H., Unciti-Broceta A., Spitzer M., White R., Tyers M., Harrington L. (2013). A yeast chemical genetic screen identifies inhibitors of human telomerase. Chem. Biol..

[145] Sengupta S., Peterson T.R., Sabatini D.M. (2010). Regulation of the mTOR complex 1 pathway by nutrients, growth factors, and stress. Mol. Cell.

[146] Bu X., Jia F., Wang W., Guo X., Wu M., Wei L. (2007). Coupled down-regulation of mTOR and telomerase activity during fluorouracil-induced apoptosis of hepatocarcinoma cells. BMC Cancer.

[147] Sundin T., Peffley D.M., Gauthier D., Hentosh P. (2012). The isoprenoid perillyl alcohol inhibits telomerase activity in prostate cancer cells. Biochimie.

[148] Sundin T., Peffley D.M., Hentosh P. (2013). Disruption of an hTERT-mTOR-RAPTOR protein complex by a phytochemical perillyl alcohol and rapamycin. Mol. Cell Biochem..

[149] Liu J.P., Chen W., Schwarer A.P., Li H. (2010). Telomerase in cancer immunotherapy. Biochim. Biophys. Acta.

[150] Ugel S., Scarselli E., Iezzi M., Mennuni C., Pannellini T., Calvaruso F., Cipriani B., De Palma R., Ricci-Vitiani L., Peranzoni E. (2010). Autoimmune B-cell lymphopenia after successful adoptive therapy with telomerase-specific T lymphocytes. Blood.

[151] Zanetti M. (2016). A second chance for telomerase reverse transcriptase in anticancer immunotherapy. Nat. Rev. Clin. Oncol..

[152] Su Z., Dannull J., Heiser A., Yancey D., Pruitt S., Madden J., Coleman D., Niedzwiecki D., Gilboa E., Vieweg J. (2003). Immunological and clinical responses in metastatic renal cancer patients vaccinated with tumor RNA-transfected dendritic cells. Cancer Res..

[153] Parkhurst M.R., Riley J.P., Igarashi T., Li Y., Robbins P.F., Rosenberg S.A. (2004). Immunization of patients with the hTERT: 540–548 peptide induces peptide-reactive T lymphocytes that do not recognize tumors endogenously expressing telomerase. Clin. Cancer Res..

[154] Vonderheide R.H., Domchek S.M., Schultze J.L., George D.J., Hoar K.M., Chen D.Y., Stephans K.F., Masutomi K., Loda M., Xia Z. (2004). Vaccination of cancer patients against telomerase induces functional antitumor CD8+ T lymphocytes. Clin. Cancer Res..

[155] Su Z., Dannull J., Yang B.K., Dahm P., Coleman D., Yancey D., Sichi S., Niedzwiecki D., Boczkowski D., Gilboa E. (2005). Telomerase mRNA-transfected dendritic cells stimulate antigen-specific CD8+ and CD4+ T cell responses in patients with metastatic prostate cancer. J. Immunol..

[156] Cortez-Gonzalez X., Zanetti M. (2007). Telomerase immunity from bench to bedside: Round one. J. Transl. Med..

[157] Brunsvig P.F., Aamdal S., Gjertsen M.K., Kvalheim G., Markowski-Grimsrud C.J., Sve I., Dyrhaug M., Trachsel S., Møller M., Eriksen J.A. (2006). Telomerase peptide vaccination: A phase I/II study in patients with non-small cell lung cancer. Cancer Immunol. Immunother..

[158] Bernhardt S.L., Gjertsen M.K., Trachsel S., Møller M., Eriksen J.A., Meo M., Buanes T., Gaudernack G. (2006). Telomerase peptide vaccination of patients with non-resectable pancreatic cancer: A dose escalating phase I/II study. Br. J. Cancer.

[159] Mavroudis D., Bolonakis I., Cornet S., Myllaki G., Kanellou P., Kotsakis A., Galanis A., Nikoloudi I., Spyropoulou M., Menez J. (2006). A phase I study of the optimized cryptic peptide TERT572y in patients with advanced malignancies. Oncology.

[160] Bolonaki I., Kotsakis A., Papadimitraki E., Aggouraki D., Konsolakis G., Vagia A., Christophylakis C., Nikoloudi I., Magganas E., Galanis A. (2007). Vaccination of patients with advanced non-small-cell lung cancer with an optimized cryptic human telomerase reverse transcriptase peptide. J. Clin. Oncol..

[161] Berntsen A., Trepiakas R., Wenandy L., Geertsen P.F., thor Straten P., Andersen M.H., Pedersen A.E., Claesson M.H., Lorentzen T., Johansen J.S. (2008). Therapeutic dendritic cell vaccination of patients with metastatic renal cell carcinoma: A clinical phase 1/2 trial. J. Immunother..

[162] Hunger R.E., Kernland Lang K., Markowski C.J., Trachsel S., Møller M., Eriksen J.A., Rasmussen A.M., Braathen L.R., Gaudernack G. (2011). Vaccination of patients with cutaneous melanoma with telomerase-specific peptides. Cancer Immunol. Immunother..

[163] Kyte J.A., Gaudernack G., Dueland S., Trachsel S., Julsrud L., Aamdal S. (2011). Telomerase peptide vaccination combined with temozolomide: A clinical trial in stage IV melanoma patients. Clin. Cancer Res..

[164] Rapoport A.P., Aqui N.A., Stadtmauer E.A., Vogl D.T., Fang H.B., Cai L., Janofsky S., Chew A., Storek J., Akpek G. (2011). Combination immunotherapy using adoptive T-cell transfer and tumor antigen vaccination on the basis of hTERT and survivin after ASCT for myeloma. Blood.

[165] Vik-Mo E.O., Nyakas M., Mikkelsen B.V., Moe M.C., Due-Tønnesen P., Suso E.M., Sæbøe-Larssen S., Sandberg C., Brinchmann J.E., Helseth E. (2013). Therapeutic vaccination against autologous cancer stem cells with mRNA-transfected dendritic cells in patients with glioblastoma. Cancer Immunol. Immunother..

[166] Fenoglio D., Traverso P., Parodi A., Tomasello L., Negrini S., Kalli F., Battaglia F., Ferrera F., Sciallero S., Murdaca G. (2013). A multi-peptide, dual-adjuvant telomerase vaccine (GX301) is highly immunogenic in patients with prostate and renal cancer. Cancer Immunol. Immunother..

[167] Staff C., Mozaffari F., Frodin J.E., Mellstedt H., Liljefors M. (2014). Telomerase (GV1001) vaccination together with gemcitabine in advanced pancreatic cancer patients. Int. J. Oncol..

[168] Davoli T., de Lange T. (2012). Telomere-driven tetraploidization occurs in human cells undergoing crisis and promotes transformation of mouse cells. Cancer Cell.

[169] Greten T.F., Forner A., Korangy F., N'Kontchou G., Barget N., Ayuso C., Ormandy L.A., Manns M.P., Beaugrand M., Bruix J. (2010). A phase II open label trial evaluating safety and efficacy of a telomerase peptide vaccination in patients with advanced hepatocellular carcinoma. BMC Cancer.

[170] Brunsvig P.F., Kyte J.A., Kersten C., Sundstrøm S., Møller M., Nyakas M., Hansen G.L., Gaudernack G., Aamdal S. (2001). Telomerase peptide vaccination in NSCLC: A phase II trial in stage III patients vaccinated after chemoradiotherapy and an 8-year update on a phase I/II trial. Clin. Cancer Res..

[171] Ellebaek E., Engell-Noerregaard L., Iversen T.Z., Froesig T.M., Munir S., Hadrup S.R., Andersen M.H., Svane I.M. (2012). Metastatic melanoma patients treated with dendritic cell vaccination, interleukin-2 and metronomic cyclophosphamide: Results from a phase II trial. Cancer Immunol. Immunother..

[172] Kotsakis A., Vetsika E.K., Christou S., Hatzidaki D., Vardakis N., Aggouraki D., Konsolakis G., Georgoulias V., Christophyllakis C., Cordopatis P. (2012). Clinical outcome of patients with various advanced cancer types vaccinated with an optimized cryptic human telomerase reverse transcriptase (TERT) peptide: Results of an expanded phase II study. Ann. Oncol..

[173] Middleton G., Silcocks P., Cox T., Valle J., Wadsley J., Propper D., Coxon F., Ross P., Madhusudan S., Roques T. (2014). Gemcitabine and capecitabine with or without telomerase peptide vaccine GV1001 in patients with locally advanced or metastatic pancreatic cancer (TeloVac): An open-label, randomised, phase 3 trial. Lancet Oncol..

[174] Mizukoshi E., Nakagawa H., Kitahara M., Yamashita T., Arai K., Sunagozaka H., Iida N., Fushimi K., Kaneko S. (2015). Phase I trial of multidrug resistance-associated protein 3-derived peptide in patients with hepatocellular carcinoma. Cancer Lett..

[175] Nemunaitis J., Tong A.W., Nemunaitis M., Senzer N., Phadke A.P., Bedell C., Adams N., Zhang Y.A., Maples P.B., Chen S. (2010). A phase I study of telomerase-specific replication competent oncolytic adenovirus (telomelysin) for various solid tumours. Mol. Ther..

[176] Keith W.N., Bilsland A., Hardie M., Evans T.R. (2004). Drug insight: Cancer cell immortality-telomerase as a target for novel cancer gene therapies. Nat. Clin. Pract. Oncol..

[177] Chen Y., Zhang Y. (2016). Functional and mechanistic analysis of telomerase: An antitumor drug target. Pharmacol. Ther..

[178] Sun L., Wang X. (2003). Effects of allicin on both telomerase activity and apoptosis in gastric cancer SGC-7901 cells. World J. Gastroenterol..

[179] Chakraborty S., Ghosh U., Bhattacharyya N.P., Bhattacharya R.K., Roy M. (2006). Inhibition of telomerase activity and induction of apoptosis by curcumin in K-562 cells. Mutat. Res..

[180] Yokoyama M., Noguchi M., Nakao Y., Ysunaga M., Yamasaki F., Iwasaka T. (2008). Antiproliferative effects of the major tea polyphenol, (−)-epigallocatechin gallate and retinoic acid in cervical adenocarcinoma. Gynecol Oncol..

[181] Ramachandran C., Fonseca H.B., Jhabvala P., Escalon E.A., Melnick S.J. (2002). Curcumin inhibits telomerase activity through human telomerase reverse transcritpase in MCF-7 breast cancer cell line. Cancer Lett..

[182] Hsin I.L., Sheu G.T., Chen H.H., Chiu L.Y., Wang H.D., Chan H.W., Hsu C.P., Ko J.L. (2010). N-acetyl cysteine mitigates curcumin-mediated telomerase inhibition through rescuing of Sp1 reduction in A549 cells. Mutat. Res..

[183] Lee J.H., Chung I.K. (2010). Curcumin inhibits nuclear localization of telomerase by dissociating the Hsp90 co-chaperone p23 from hTERT. Cancer Lett..

[184] Singh M., Singh N. (2009). Molecular mechanism of curcumin induced cytotoxicity in human cervical carcinoma cells. Mol. Cell Biochem..

[185] Mukherjee Nee Chakraborty S., Ghosh U., Bhattacharyya N.P., Bhattacharya R.K., Dey S., Roy M. (2007). Curcumin-induced apoptosis in human leukemia cell HL-60 is associated with inhibition of telomerase activity. Mol. Cell Biochem..

[186] Berletch J.B., Liu C., Love W.K., Andrews L.G., Katiyar S.K., Tollefsbol T.O. (2008). Epigenetic and genetic mechanisms contribute to telomerase inhibition by EGCG. J. Cell Biochem..

[187] Li Y., Liu L., Andrews L.G., Tollefsbol T.O. (2009). Genistein depletes telomerase activity through cross-talk between genetic and epigenetic mechanisms. Int. J. Cancer.

[188] Meeran S.M., Patel S.N., Chan T.H., Tollefsbol T.O. (2011). A novel prodrug of epigallocatechin-3-gallate: Differential epigenetic hTERT repression in human breast cancer cells. Cancer Prev. Res. (Phila).

[189] Moon D.O., Kang S.H., Kim K.C., Kim M.O., Choi Y.H., Kim G.Y. (2010). Sulforaphane decreases viability and telomerase activity in hepatocellular carcinoma Hep3B cells through the reactive oxygen species-dependent pathway. Cancer Lett..

[190] Mittal A., Pate M.S., Wylie R.C., Tollefsbol T.O., Katiyar S.K. (2004). EGCG down-regulates telomerase in human breast carcinoma MCF-7 cells, leading to suppression of cell viability and induction of apoptosis. Int. J. Oncol..

[191] Shapira S., Granot G., Mor-Tzuntz R., Raanani P., Uziel O., Lahav M., Shpilberg O. (2012). Second-generation tyrosine kinase inhibitors reduce telomerase activity in K562 cells. Cancer Lett..

[192] Mor-Tzuntz R., Uziel O., Shpilberg O., Lahav J., Raanani P., Bakhanashvili M., Rabizadeh E., Zimra Y., Lahav M., Granot G. (2010). Effect of imatinib on the signal transduction cascade regulating telomerase activity in K562 (BCR-ABL-positive) cells sensitive and resistant to imatinib. Exp. Hematol..

[193] Moon D.O., Kim M.O., Heo M.S., Lee J.D., Choi Y.H., Kim G.Y. (2009). Gefitinib induces apoptosis and decreases telomerase activity in MDA-MB-231 human breast cancer cells. Arch. Pharm. Res..

[194] Zhang Y., Sun M., Shi W., Yang Q., Chen C., Wang Z., Zhou X. (2015). Arsenic trioxide suppresses transcription of hTERT through down-regulation ofmultiple transcription factors in HL-60 leukemia cells. Toxicol. Lett..

[195] Zhang X., Li B., de Jonge N., Bjorkholm M., Xu D. (2015). The DNA methylation inhibitor induces telomere dysfunction and apoptosis of leukemia cells that is attenuated by telomerase over-expression. Oncotarget.

[196] Kanzawa T., Germano I.M., Kondo Y., Ito H., Kyo S., Kondo S. (2003). Inhibition of telomerase activity in malignant glioma cells correlates with their sensitivity to temozolomide. Br. J. Cancer.

[197] Gan Y., Lu J., Yeung B.Z., Cottage C.T., Wientjes M.G., Au J.L. (2015). Pharmacodynamics of telomerase inhibition and telomere shortening by noncytotoxic suramin. AAPS J..

[198] He H., Xia H.H., Wang J.D., Gu Q., Lin M.C., Zou B., Lam S.K., Chan A.O., Yuen M.F., Kung H.F. (2006). Inhibition of human telomerase reverse transcriptase by nonsteroidal antiinflammatory drugs in colon carcinoma. Cancer.

[199] Zhao Y.-Q., Feng H.-W., Jia T., Chen X.-M., Zhang H., Xu A.-T., Zhang H.L., Fan X.-L. (2014). Antiproliferative effects of celecoxib in Hep-2 cells through telomerase inhibition and induction of apoptosis. Asian Pac. J. Cancer Prev..

[200] Rashid-Kolvear F., Taboski M.A., Nguyen J., Wang D.Y., Harrington L.A., Done S.J. (2011). Troglitazone suppresses telomerase activity independently of PPARgamma in estrogen-receptor negative breast cancer cells. BMC Cancer.

[201] Kiran K.G., Palaniswamy M., Angayarkanni J. (2015). Human telomerase inhibitors from microbial source. World J. Microbiol. Biotechnol..

[202] Li C.T., Hsiao Y.M., Wu T.C., Lin Y.W., Yeh K.T., Ko J.L. (2011). Vorinostat, SAHA, represses telomerase activity via epigenetic regulation of telomerase reverse transcriptase in non–small cell lung cancer cells. J. Cell Biochem..

[203] Zhao Y.M., Zhou Q., Xu Y., Lai X.Y., Huang H. (2008). Antiproliferative effect of rapamycin on human T-cell leukemia cell line Jurkat by cell cycle arrest and telomerase inhibition. Acta Pharmacol. Sin..

[204] Woo H.J., Choi Y.H. (2005). Growth inhibition of A549 human lung carcinoma cells by beta-lapachone through induction of apoptosis and inhibition of telomerase activity. Int. J. Oncol..

[205] Burger A.M., Double J.A., Newell D.R. (1997). Inhibition of telomerase activity by cisplatin in human testicular cancer cells. Eur. J. Cancer.

[206] Leon-Blanco M.M., Guerrero J.M., Reiter R.J., Calvo J.R., Pozo D. (2003). Melatonin inhibits telomerase activity in the MCF-7 tumor cell line both in vivo and in vitro. J. Pineal. Res..

[207] Holohan B., Hagiopian M.M., Lai T.P., Huang E., Friedman D.R., Wright W.E., Shay J.W. (2015). Perifosine as a potential novel anti-telomerase therapy. Oncotarget.

[208] Baoping Y., Guoyong H., Jieping Y., Zongxue R., Hesheng L. (2004). Cyclooxygenase-2 inhibitor nimesulide suppresses telomerase activity by blocking Akt/PKB activation in gastric cancer cell line. Dig. Dis. Sci..

[209] Kim N.-H., Park H.J., Oh M.-K., Kim I.-S. (2013). Antiproliferative effect of gold (I) compound auranofin through inhibition of STAT3 and telomerase activity in MDA-MB 231 human breast cancer cells. BMB Rep..

[210] Khorramizadeh M.R., Saadat F., Vaezzadeh F., Safavifar F., Bashiri H., Jahanshiri Z. (2007). Suppression of telomerase activity by pyrimethamine: Implication to cancer. Iran Biomed. J..

[211] Brown T., Sigurdson E., Rogatko A., Broccoli D. (2003). Telomerase inhibition using azidothymidine in the HT-29 colon cancer cell line. Ann. Surg. Oncol..

[212] Gao S., Yu B.P., Li Y., Dong W.G., Luo H.S. (2003). Antiproliferative effect of octreotide on gastric cancer cells mediated by inhibition of Akt/PKB and telomerase. World J. Gastroenterol..

[213] Yamakuchi M., Nakata M., Kawahara K., Kitajima I., Maruyama I. (1997). New quinolones, ofloxacin and levofloxacin, inhibit telomerase activity in transitional cell carcinoma cell lines. Cancer Lett..

[214] Sun H., Xiang J., Li Q., Liu Y., Li L., Shang Q., Xu G., Tang Y. (2012). Recognize three different human telomeric G-quadruplex conformations by quinacrine. Analyst.

[215] Ci X., Li B., Ma X., Kong F., Zheng C., Björkholm M., Jia J., Xu D. (2015). Bortezomibmediated down-regulation of telomerase and disruption of telomere homeostasis contributes to apoptosis of malignant cells. Oncotarget.

[216] Kato M., Nakayama M., Agata M., Yoshida K. (2013). Gene expression levels of human shelterin complex and shelterin-associated factors regulated by the topoisomerase II inhibitors doxorubicin and etoposide in human cultured cells. Tumour Biol..

[217] Zhang B., Qian D., Ma H.H., Jin R., Yang P.X., Cai M.Y., Liu Y.H., Liao Y.J., Deng H.X., Mai S.J. (2012). Anthracyclines disrupt telomere maintenance by telomerase through inducing PinX1 ubiquitination and degradation. Oncogene.

[218] Mimeault M., Hauke R., Mehta P.P., Batra S.K. (2007). Recent advances on cancer stem/progenitor cell research: Therapeutic implications for overcoming resistance to the most aggressive cancers. J. Mol. Cell Med..

[219] Mimeault M., Batra S.K. (2008). Recent advances on the development of novel anti-cancer drugs targeting cancer stem/progenitor cells. Drug Dev. Res..

[220] Tang J.Y., So P.L., Epstein E.H. (2006). Novel Hedgehog pathway targets against basal cell carcinoma. Toxicol. Appl. Pharmacol..

[221] Singh B., Schneider M., Knyazev P., Ullrich A. (2009). UV-induced EGFR signal transactivation is dependent on proligand shedding by activated metalloproteases in skin cancer cell lines. Int. J. Cancer.

[222] Stecca B., Mas C., Clement V., Zbinden M., Correa R., Piguet V., Beermann F., Ruiz I., Altaba A. (2007). Melanomas require HEDGEHOG-GLI signaling regulated by interactions between GLI1 and the RAS-MEK/AKT pathways. Proc. Natl. Acad. Sci. USA.

[223] Hoffmeyer K., Raggioli A., Rudloff S., Anton R., Hierholzer A., Del Valle I., Hein K., Vogt R., Kemler R. (2012). Wnt/β-catenin signaling regulates telomerase in stem cells and cancer cells. Science.

[224] Calado R.T., Young N.S. (2008). Telomere maintenance and human bone marrow failure. Blood.

[225] Yamamoto K., Nihrane A., Aglipay J., Sironi J., Arkin S., Lipton J.M., Ouchi T., Liu J.M. (2008). Upregulated ATM gene expression and activated DNA crosslink-induced damage response checkpoint in Fanconi anemia: Implications for carcinogenesis. Mol. Med..

[226] Meshorer E., Gruenbaum Y. (2008). Gone with the Wnt/Notch: Stem cells in laminopathies, progeria, and aging. J. Cell Biol..

[227] Scaffidi P., Misteli T. (2008). Lamin A-dependent misregulation of adult stem cells associated with accelerated ageing. Nat. Cell Biol..

[228] Bergoglio V., Magnaldo T. (2006). Nucleotide excision repair and related human diseases. Genome Dyn..

[229] Stout G.J., Blasco M.A. (2009). Genetic dissection of the mechanisms underlying telomereassociated diseases: Impact of the TRF2 telomeric protein on mouse epidermal stem cells. Dis. Model Mech..

[230] Aubert G., Lansdorp P.M. (2008). Telomeres and aging. Physiol. Rev..

[231] Mason P.J., Wilson D.B., Bessler M. (2005). Dyskeratosis congenital—A disease of dysfunctional telomere maintenance. Curr. Mol. Med..

[232] Kenyon J., Gerson S.L. (2007). The role of DNA damage repair in aging of adult stem cells. Nucleic Acids Res..

[233] Nijnik A., Woodbine L., Marchetti C., Dawson S., Lambe T., Liu C., Rodrigues N.P., Crockford T.L., Cabuy E., Vindigni A. (2007). DNA repair is limiting for haematopoietic stem cells during ageing. Nature.

[234] Chigancas V., Lima-Bessa K.M., Stary A., Menck C.F., Sarasin A. (2008). Defective transcription/repair factor IIH recruitment to specific UV lesions in trichothiodystrophy syndrome. Cancer Res..

[235] Budiyanto A., Bito T., Kunisada M., Ashida M., Ichihashi M., Ueda M. (2003). Inhibition of the epidermal growth factor receptor suppresses telomerase activity in HSC-1 human cutaneous squamous cell carcinoma cells. J. Invest. Dermatol..

[236] Horn S., Figl A., Rachakonda P.S., Fischer C., Sucker A., Gast A., Kadel S., Moll I., Nagore E., Hemminki K. (2013). TERT promoter mutations in familial and sporadic melanoma. Science.

[237] Baur J.A., Sinclair D.A. (2006). Therapeutic potential of resveratrol: The in vivo evidence. Nat. Rev. Drug Discov..

[238] Athar M., Back J.H., Tang X., Kim K.H., Kopelovich L., Bickers D.R., Kim A.L. (2007). Resveratrol: A review of preclinical studies for human cancer prevention. Toxicol. Appl. Pharmacol..

[239] Shay J.W., Wright W.E. (2010). Telomeres and telomerase in normal and cancer stem cells. FEBS Lett..

[240] Hahn W.C., Counter C.M., Lundberg A.S., Beijersbergen R.L., Brooks M.W., Weinberg R.A. (1999). Creation of human tumor cells with defined genetic elements. Nature.

[241] Braig M., Lee S., Loddenkemper C., Rudolph C., Peters A.H., Schlegelberger B., Stein H., Dörken B., Jenuwein T., Schmitt C.A. (2005). Oncogene-induced senescence as an initial barrier in lymphoma development. Nature.

[242] Chen Z., Trotman L.C., Shaffer D., Lin H.K., Dotan Z.A., Niki M., Koutcher J.A., Scher H.I., Ludwig T., Gerald W. (2005). Crucial role ofp53-dependent cellular senescence in suppression of Pten-deficient tumorigenesis. Nature.

[243] Collado M., Serrano M. (2006). The power and the promise of oncogene-induced senescence markers. Nat. Rev. Cancer.

[244] Xue W., Zender L., Miething C., Dickins R.A., Hernando E., Krizhanovsky V., Cordon-Cardo C., Lowe S.W. (2007). Senescence and tumour clearance is triggered by p53 restoration in murineliver carcinomas. Nature.

[245] Ventura A., Kirsch D.G., McLaughlin M.E., Tuveson D.A., Grimm J., Lintault L., Newman J., Reczek E.E., Weissleder R., Jacks T. (2007). Restoration of p53 function leads to tumour regression in vivo. Nature.

[246] Ewald J.A., Desotelle J.A., Wilding G., Jarrard D.F. (2010). Therapy-induced senescence in cancer. J. Natl. Cancer Inst..

[247] Te Poele R.H., Okorokov A.L., Jardine L., Cummings J., Joel S.P. (2002). DNA damage is able to induce senescence in tumor cells in vitro and in vivo. Cancer Res..

[248] Rudolph K.L., Millard M., Bosenberg M.W., DePinho R.A. (2001). Telomere dysfunction and evolution of intestinal carcinoma in mice and humans. Nat. Genet..

[249] Chin L., Artandi S.E., Shen Q., Tam A., Lee S.-L., Gottlieb G.J., Greider C.W., DePinho R.A. (1999). p53 deficiency rescues the adverse effects of telomere loss and cooperates with telomere dysfunction to accelerate carcinogenesis. Cell.

[250] Artandi S.E., Chang S., Lee S.L., Alson S., Gottlieb G.J., Chin L., DePinho R.A. (2000). Telomere dysfunction promotes non-reciprocal translocations and epithelial cancers in mice. Nature.

[251] O’Sullivan J.N., Bronner M.P., Brentnall T.A., Finley J.C., Shen W.T., Emerson S., Emond M.J., Gollahon K.A., Moskovitz A.H., Crispin D.A. (2002). Chromosomal instability in ulcerative colitis is related to telomere shortening. Nat. Genet..

[252] Romanov S.R., Kozakiewicz B.K., Holst C.R., Stampfer M.R., Haupt L.M., Tlsty T.D. (2001). Normal human mammary epithelial cells spontaneously escape senescence and acquire genomic changes. Nature.

[253] Seger Y.R., García-Cao M., Piccinin S., Cunsolo C.L., Doglioni C., Blasco M.A., Hannon G.J., Maestro R. (2002). Transformation of normal human cells in the absence of telomerase activation. Cancer Cell.

[254] Tomas-Loba A., Flores I., Fernandez-Marcos P.J., Cayuela M.L., Maraver A., Tejera A., Borras C., Matheu A., Klatt P., Flores J.M. (2008). Telomerase reverse transcriptase delays aging in cancer resistant mice. Cell.

[255] Blasco M.A. (2005). Telomeres and human disease: Ageing, cancer and beyond. Nat. Rev. Genet..

[256] Gonzalez-Suarez E., Geserick C., Flores J.M., Blasco M.A. (2005). Antagonistic effects of telomerase on cancer and aging in K5-mTert transgenic mice. Oncogene.

[257] Bernardes de Jesus B., Vera E., Schneeberger K., Tejera A.M., Ayuso E., Bosch F., Blasco M.A. (2012). Telomerase gene therapy in adult and old mice delays aging and increases longevity without increasing cancer. EMBO Mol. Med..

